# Targets of histone H3 lysine 9 methyltransferases

**DOI:** 10.3389/fcell.2022.1026406

**Published:** 2022-12-06

**Authors:** Aidan J. Levinsky, Gregor McEdwards, Nasha Sethna, Mark A. Currie

**Affiliations:** ^1^ Department of Biology, University of Toronto Mississauga, Mississauga, ON, Canada; ^2^ Department of Cell and Systems Biology, University of Toronto, Toronto, ON, Canada

**Keywords:** methyltransferase, methylation, histone, chromatin, heterochromatin

## Abstract

Histone H3 lysine 9 di- and trimethylation are well-established marks of constitutively silenced heterochromatin domains found at repetitive DNA elements including pericentromeres, telomeres, and transposons. Loss of heterochromatin at these sites causes genomic instability in the form of aberrant DNA repair, chromosome segregation defects, replication stress, and transposition. H3K9 di- and trimethylation also regulate cell type-specific gene expression during development and form a barrier to cellular reprogramming. However, the role of H3K9 methyltransferases extends beyond histone methylation. There is a growing list of non-histone targets of H3K9 methyltransferases including transcription factors, steroid hormone receptors, histone modifying enzymes, and other chromatin regulatory proteins. Additionally, two classes of H3K9 methyltransferases modulate their own function through automethylation. Here we summarize the structure and function of mammalian H3K9 methyltransferases, their roles in genome regulation and constitutive heterochromatin, as well as the current repertoire of non-histone methylation targets including cases of automethylation.

## 1 Introduction

Lysine methylation is a dynamic posttranslational modification (PTM) that regulates protein structure and function in all three domains of life (Manzur and Zhou, 2005; Paik et al., 2007). The human genome is predicted to contain over one hundred protein lysine methyltransferases and almost two dozen demethylases that display a variety of substrate and product specificities (Shi and Tsukada, 2013; Husmann and Gozani, 2019). The first of these lysine methyltransferases to be identified was mammalian suppressor of variegation 3–9 homologue 1 (SUV39H1), *Drosophila melanogaster* suppressor of variegation 3–9 Su(var)3–9, and its *Schizosaccharomyces pombe* homolog, cryptic loci regulator 4 (Clr4), which produce histone H3 lysine 9 di- and trimethylation (H3K9me2 and H3K9me3) (Rea et al., 2000; Czermin et al., 2001; Nakayama et al., 2001). Since this discovery, many other methyltransferases have also been shown to methylate histones at various sites, which regulates genome packaging, organization, and functions including gene expression, DNA damage response, and DNA replication (Husmann and Gozani, 2019).

Histone proteins are highly conserved basic proteins that along with other non-histone proteins package and organize the eukaryotic genome into a complex called chromatin. The fundamental unit of chromatin, the nucleosome, is composed of approximately 147 base pairs of DNA wrapped around a histone octamer containing two copies of each of the core histones—H2A, H2B, H3, and H4 (Kornberg, 1974; Luger et al., 1997). Observations made by Emil Heitz in the 1920s established that there are two distinct types of chromatin for which he coined the terms ‘heterochromatin’ and ‘euchromatin,’ referring to condensed darkly stained chromosomal regions and decondensed light staining regions, respectively (Heitz, 1928). Euchromatin is associated with active transcription, whereas heterochromatin is predominantly transcriptionally repressed (Allis and Jenuwein, 2016). Heterochromatin clusters into distinct compartments within the nucleus including the perinucleolar region and at the nuclear periphery in a variety of different species including *S. pombe*, *D. melanogaster, Caenorhabditis elegans*, mouse, and humans (Funabiki et al., 1993; Horsley et al., 1996; Minc et al., 1999; Pickersgill et al., 2006; Guelen et al., 2008; Ikegami et al., 2010; Peric-Hupkes et al., 2010). These localizations and clustering are mediated by protein-protein interactions as well as phase separation (Olins et al., 2010; Brachner and Foisner, 2011; Towbin et al., 2012; Poleshko et al., 2013; Solovei et al., 2013; Wong et al., 2014; Larson et al., 2017; Strom et al., 2017). H3K9me2 and H3K9me3 are the hallmarks of heterochromatin conserved from fission yeast to humans (Allshire and Madhani, 2018). Traditionally, H3K9 methylation (H3K9me) is associated with the silencing of repetitive DNA sequences including pericentromere and subtelomere repeats and transposable elements in order to maintain genome stability (Janssen et al., 2018). This is referred to as constitutive heterochromatin because these domains remain methylated and silent throughout the cell cycle and development in most cell types. However, more recently, H3K9me has been reported to silence protein-coding genes in a cell type-specific manner throughout development, which is called facultative heterochromatin (Padeken et al., 2022). In addition to its role in establishing cell type-specific gene expression programs, H3K9me also helps stabilize cell fate decisions and maintain cell identity (Becker et al., 2016; Padeken et al., 2022). H3K9me coats critical genomic binding sites for master regulator proteins in terminally differentiated cells, which reduces binding of these master regulators and hinders cellular reprogramming of induced pluripotent stem (iPS) cells and somatic cell nuclear transfer (SCNT) (Shi et al., 2008; Onder et al., 2012; Soufi et al., 2012; Sridharan et al., 2013; Matoba et al., 2014; Becker et al., 2016; Liu et al., 2018).

Mammals have six well characterized H3K9 methyltransferases including SUV39H1 and SUV39H2, SET domain bifurcated 1 and 2 (SETDB1 and SETDB2), G9a, and G9a-like protein (GLP). A compound mutant eliminating the function of all six of these methyltransferases in mouse embryonic fibroblasts was recently generated (Montavon et al., 2021). In these cells, a complete loss of all H3K9 methylation states, decondensation of heterochromatin, additive derepression of a wide variety of repeat elements, genome instability, and loss of heterochromatin compartmentalization was observed (Montavon et al., 2021). Partial overlapping functions between mammalian H3K9 methyltransferases have been identified in this and other studies (Montavon et al., 2021; Fritsch et al., 2010; Bulut-Karslioglu et al., 2014; García-Cao et al., 2004; Gauchier et al., 2019; Maksakova et al., 2013; Liu et al., 2014; di Giacomo et al., 2014). These redundancies combined with technical challenges involved with mapping genomic H3K9me complicate studies of H3K9me in mammalian systems (Montavon et al., 2021; Fritsch et al., 2010; Bulut-Karslioglu et al., 2014; García-Cao et al., 2004; Gauchier et al., 2019; Maksakova et al., 2013; Liu et al., 2014; di Giacomo et al., 2014; Becker et al., 2017). As a result, much of what we know about the role and regulation of H3K9me has come from studies in simpler model organisms with fewer H3K9 methyltransferases including *S. pombe*, *D. melanogaster*, and *C. elegans* (Elgin and Reuter, 2013; Holoch and Moazed, 2015; Ahringer and Gasser, 2018; Allshire and Madhani, 2018).

However, H3K9 is not the only target of H3K9 methyltransferases. They methylate a variety of other histone and non-histone targets, which have important functional consequences in gene regulation. SUV39H1 and SUV39H2 primarily methylate other chromatin modifying proteins (Zhang et al., 2011; Piao et al., 2015; Kudithipudi et al., 2017). SETDB1 methylates proteins involved in proliferation and cell cycle regulation including AKT and mutant p53, while G9a/GLP regulates various transcription factors (TFs) as well as several hypoxia stress response factors (Feldman et al., 2006; Pless et al., 2008; Lee et al., 2010; Lee et al., 2011; Ling et al., 2012; Choi et al., 2014; Bao et al., 2018). SUV39H2 and G9a/GLP have also been shown to regulate their own functions through automethylation (Chin et al., 2007; Sampath et al., 2007; Piao et al., 2016; Poulard et al., 2017; Iglesias et al., 2018).

In this review, we will provide a synopsis of the structural and biochemical characteristics that define the main classes of H3K9 methyltransferases in mammals. We will describe the canonical role H3K9me plays in silencing constitutive heterochromatin domains and the maintenance of genome stability along with its more recently appreciated function in the regulation of cell type-specific gene expression. Lastly, we will highlight the regulation of non-histone targets by H3K9 methyltransferase-mediated methylation and automethylation.

### 1.1 Structure and domain architecture of H3K9 methyltransferases

**FIGURE 1:**
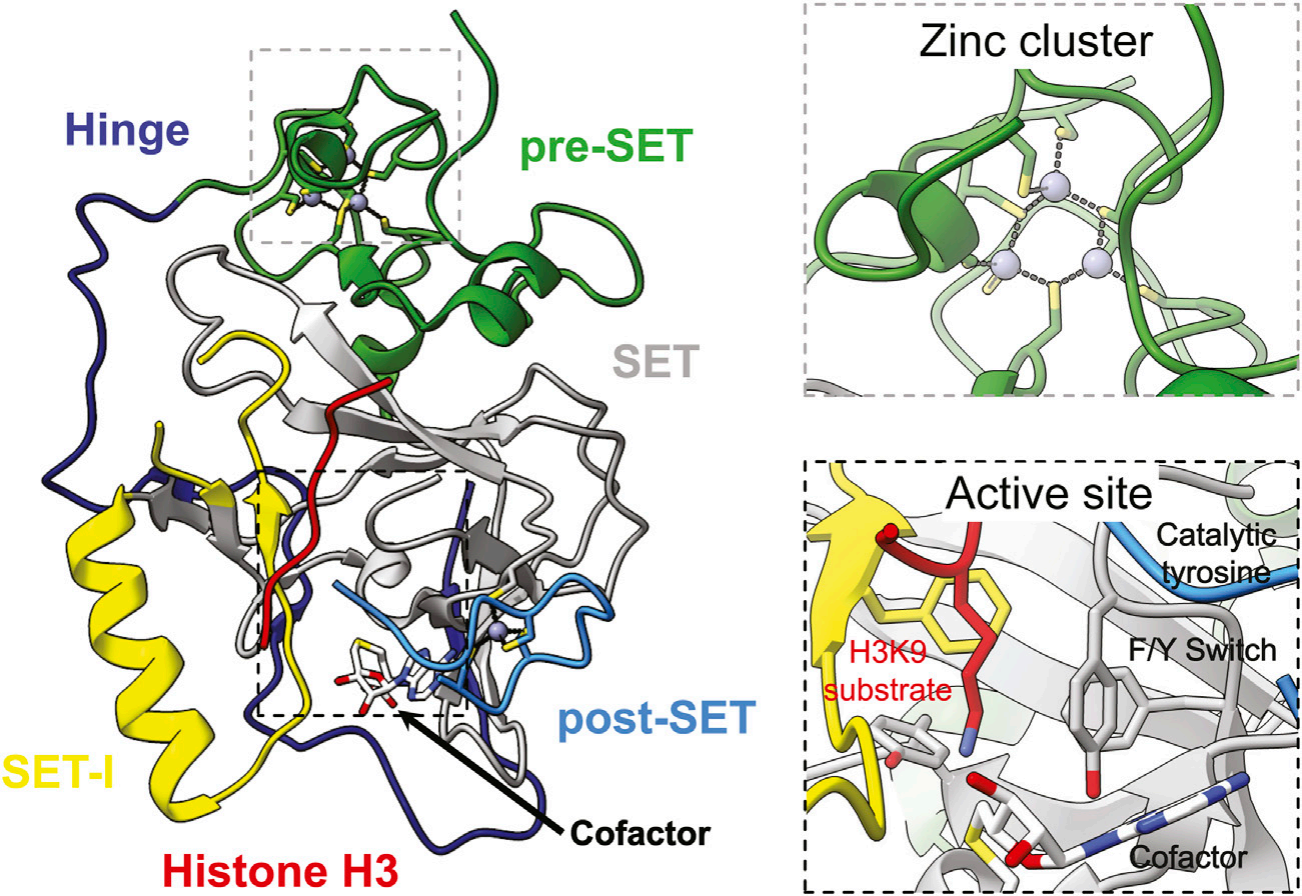
Structure of the conserved core catalytic SET domain of H3K9 methyltransferases. Structure of the first SET domain to be determined, Dim-5 an H3K9 methyltransferase from *Neurospora crassa*, in complex with cofactor (white) and histone peptide substrate (red) (PDB ID 1PEG) (left). Close up of conserved cysteines and triangular zinc cluster of the pre-SET domain (top right). Close up of active site (bottom right). The Hinge, pre-SET, SET, SET-I, and post-SET domains are coloured dark blue, green, grey, yellow, and light blue, respectively. Zinc atoms are depicted as dark grey spheres.

**FIGURE 2:**
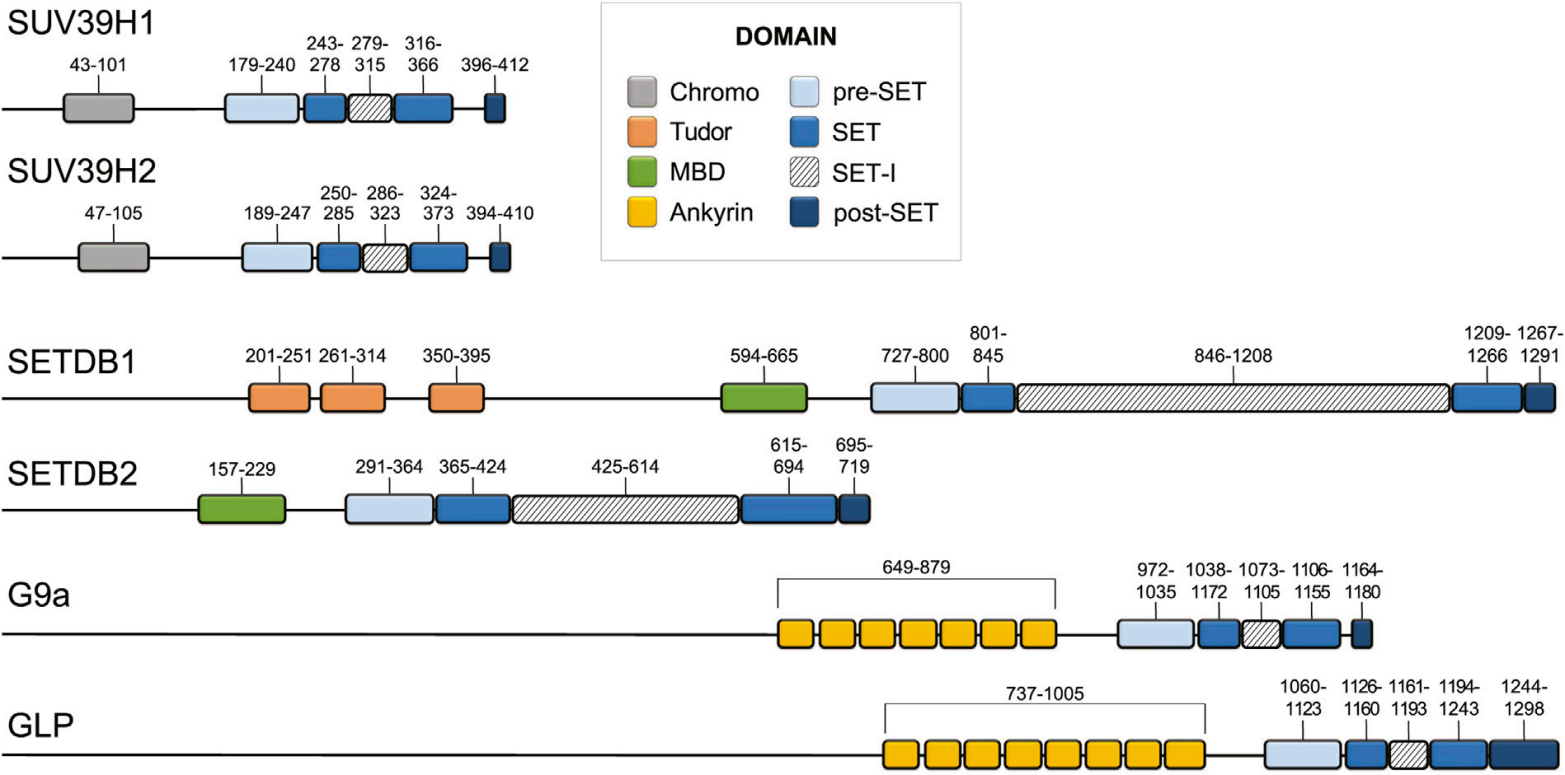
Domain architecture of the main human H3K9 methyltransferases. Domain architecture of SUV39H1, SUV39H2, SETDB1, SETDB2, G9a, and GLP (Apweiler et al., 2004; Wu et al., 2010; Torrano et al., 2019; Weirich et al., 2021). Domain boundaries are listed above each domain with chromodomain, pre-SET, SET, SET-I, post-SET, Tudor, MBD, and Ankyrin repeat domains coloured grey, light blue, blue, diagonal hatching, dark blue, orange, green, and yellow, respectively.

**FIGURE 3:**
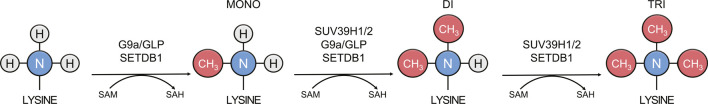
Methylation states catalyzed by main mammalian H3K9 methyltransferases. SUV39H1, SUV39H2, SETDB1, SETDB2, G9a, and GLP transfer methyl groups (red) from cofactor SAM to the epsilon nitrogen (blue) of lysine residues to create mono-, di-, and trimethyllysine.

All lysine methyltransferases, except for DOT1-like methyltransferases, contain a catalytic SET domain named after the three founding members Su(var)3–9, Enhancer-of-zeste, and Trithorax (Jones and Gelbart, 1993; Tschiersch et al., 1994; Stassen et al., 1995; Jenuwein et al., 1998; Min et al., 2003). The core SET domain structure is comprised of two non-contiguous regions that span approximately 130 amino acids (Figure 1)(Dillon et al., 2005). The N-terminal and C-terminal regions are both highly conserved and consist of a short helix and three or four short *ß*-strands that adopts a *ß*-sheet fold pseudo-knot-type structure (Figure 1) (Dillon et al., 2005). An insert region, referred to as SET-I, joins the two-halves of the SET domain and plays important roles in substrate recognition and enzyme regulation (Figure 1) (Qian and Zhou, 2006; Jiao and Liu, 2015; Ishimoto et al., 2016; Li et al., 2016; Sun and Fang, 2016). The size and structure of SET-I varies greatly depending on the methyltransferase: SUV39H2 SET-I consists of 37 amino acids that form a helix followed by a short loop (Wu et al., 2010); whereas, SETDB1 has a 362 amino acid SET-I that is predicted to contain a mixed structure. The canonical SET domain is flanked by both pre- and post-SET domains in the SUV39-family of methyltransferases, which includes all H3K9 methyltransferases (Figures 1, 2) (Dillon et al., 2005). The pre-SET domain is composed of random coil with nine invariant cysteines that coordinate three zinc ions in a triangular cluster (Figure 1) (Dillon et al., 2005). Six of the cysteines are involved in the coordination of a single zinc atom with the remaining three cysteines coordinating two zinc atoms each (Dillon et al., 2005). The post-SET domain is also composed of random coil (Figure 1) (Dillon et al., 2005). Three conserved cysteines from the post-SET domain and a fourth in the knot-like structure of the SET domain close to the active site coordinate a single zinc atom that is required for methyltransferase activity (Figure 1) (Zhang et al., 2002; Dillon et al., 2005). Proper folding of the post-SET domain is required for methyltransferase activity as mutating these invariant cysteines abolish enzyme function (Rea et al., 2000; Schultz et al., 2002; Zhang et al., 2002). The conserved catalytic tyrosine is found at the C-terminus of SET domain with the methyl donor s-adenosyl-l-methionine (SAM) sandwiched between the core SET domain, SET-I, and the post-SET domain (Figure 1) (Qian and Zhou, 2006). Product specificity is dictated by a phenylalanine/tyrosine switch site located two amino acids N-terminal to the catalytic tyrosine (Collins et al., 2005; Couture et al., 2008). There are four possible lysine methylation states—unmethylated, monomethylated, dimethylated, and trimethylated—each of which represent distinct modifications associated with unique biological consequences (Figure 3) (Black et al., 2012). Enzymes with a phenylalanine in the switch position catalyze di- and tri-methylation, whereas tyrosine in the switch position restricts the enzymes to mono- and dimethylation (Collins et al., 2005; Couture et al., 2008).

Of the six mammalian SUV39-family H3K9 methyltransferases: G9a and GLP generate mono- and dimethylation and SUV39H1, SUV39H2, SETDB1, and SETDB2 catalyze di- and trimethylation, with the majority of trimethylation coming from SUV39H1 and SUV39H2 *in vivo* (Wu et al., 2010; Montavon et al., 2021) (Figure 3). These enzymes display differences in the SET-I region and in their complement of N-terminal domains (Marmorstein, 2003; Xiao et al., 2003) (Figure 2). In addition to a catalytic “writer” domain, some histone methyltransferases also have what are referred to as “reader” domains, which recognize and recruit the enzyme to regions of the genome enriched in a particular modification (Hyun et al., 2017). The “read/write” mechanism refers to a particular situation when the writer domain produces a modification that is recognized by a reader domain on the same protein, which creates a positive feedback loop that reinforces a chromatin state (Zhang et al., 2015). In addition to the “reader” domains, H3K9 methyltransferases are also recruited via TFs, non-coding RNA, the RNAi pathway, and m^6^A RNA (Schultz et al., 2002; Jia et al., 2004; Porro et al., 2014; Holoch and Moazed, 2015; Scarola et al., 2015; Johnson et al., 2017; Shirai et al., 2017; Velazquez Camacho et al., 2017; Ahringer and Gasser, 2018; Tie et al., 2018; Erdmann and Picard, 2020; Chelmicki et al., 2021; Liu et al., 2021; Xu et al., 2021; Wei et al., 2022). SUV39H1 and SUV39H2, two highly related H3K9 methyltransferases, have an N-terminal chromodomain connected to their catalytic SET domains via a flexible hinge region (Figure 2) (Melcher et al., 2000; O’Carroll et al., 2000). The chromodomain is 40–50 residues composed of a short alpha helix packed against three antiparallel beta strands that recognize and recruit SUV39H1 and SUV39H2 to H3K9me via a conserved aromatic cage (Jacobs and Khorasanizadeh, 2002; Wang et al., 2012). The chromodomain of SUV39H1 and the mouse-specific N-terminal 81 amino acid basic domain of SUV39H2 also bind RNA transcribed from major satellite repeats, which provides a mechanism to recruit SUV39H1 and SUV39H2 to transcribed repeat sequences (Johnson et al., 2017; Shirai et al., 2017; Velazquez Camacho et al., 2017). The chromodomain and linker region between the chromodomain and pre-SET domain of Clr4 has been shown to interact with the nucleosome core (Akoury et al., 2019). G9a reads H3K9me1 and H3K9me2 through N-terminal ankyrin repeat domains (Figure 2) (Collins et al., 2008). GLP is a paralog of G9a which shares 70% sequence similarity. The greatest sequence divergence between G9a and GLP is in the N-terminus of the protein at the glutamic acid rich domain of G9a, which contains a series of repeated aspartic and glutamic acid residues in GLP. Although G9a and GLP are capable of forming both homo and heterodimers via their SET domains, the heterodimer is the more active form of the enzyme with greater reading and writing capabilities *in vitro* (Sanchez et al., 2021). However, the significance of this observation remains to be tested *in vivo*. SETDB1 and SETDB2 contain three N-terminal Tudor domains and a methyl-CpG binding domain (MBD) domain (Figure 2) (Jurkowska et al., 2017). The Tudor domains form complexes with other regulators that are vital for transcriptional repression and cooperate to recognize histone tails bearing both H3K14 acetylation and H3K9 methylation (Yang et al., 2003; Li et al., 2006; Jurkowska et al., 2017). The MBD domain possesses two arginine residues that facilitate DNA binding and link H3K9 trimethylation with DNA methylation via MBD interaction with DNA methyltransferase 3 in organisms that have DNA methylation (Li et al., 2006; Chen et al., 2017). However, the MBD domain is conserved in species that lack DNA methylation including *D. melanogaster* and *C. elegans*, which suggests that the MBD domain may also possesses DNA methylation-independent functions. The N-terminus of SETDB1 also contains a binding site for activating transcriptional factor 7-interacting protein 1 (ATF7IP), which is a conserved cofactor of SETDB1 that stimulates SETDB1 methyltransferase activity and is required for its nuclear localization and chromatin association (Wang et al., 2003; Timms et al., 2016; Mutlu et al., 2018; Delaney et al., 2019; Osumi et al., 2019; Tsusaka et al., 2019).

## 2 Constitutive heterochromatin and the regulation of repetitive DNA elements

About half of the human genome is composed of repetitive DNA elements including centromeres and pericentromeric regions, telomeres and subtelomeres, transposons, and ribosomal DNA (rDNA) that are packaged into constitutive heterochromatin (Lander et al., 2001; Venter et al., 2001). H3K9me and silencing of these regions is critical for maintaining genome stability by protecting telomeres and preventing chromosome segregation defects, recombination, and transposition (Ekwall et al., 1996; Nonaka et al., 2002; Slotkin and Martienssen, 2007; Peng and Karpen, 2009). The role of H3K9me and H3K9 methyltransferases is best understood in constitutive heterochromatin. Mammalian H3K9 methyltransferases play both distinct and overlapping functions in the silencing of these repetitive elements. A multimeric complex containing SUV39H1, G9a, GLP, and SETDB1 has been reported, which may help explain some of the redundancies (Fritsch et al., 2010). In SUV39H1 or G9a knockout cells the remaining methyltransferase components of the complex are destabilized, which results in an overall reduction in their protein levels (Fritsch et al., 2010). SUV39H1 and SUV39H2 target pericentromeric repeats, telomeres, class II endogenous retroviruses (ERVs), and long interspersed nuclear elements (LINEs) (García-Cao et al., 2004; Martens et al., 2005; Bulut-Karslioglu et al., 2014). SUV39H1 also represses non-transcribed rDNA repeats (Murayama et al., 2008). However, in the absence of SUV39H1 and SUV39H2, low levels of H3K9me3 persist at telomeres and LINE elements that are SETDB1-dependent (García-Cao et al., 2004; Bulut-Karslioglu et al., 2014; Gauchier et al., 2019). Endoderm-specific conditional SETDB1 knockout mice display only a modest reduction in H3K9me3 levels (Nicetto et al., 2019). The conditional knockout of SETDB1, SUV39H1, and SUV39H2 together causes a substantial decrease in H3K9me3 and marked derepression of nonhepatic genes in mouse livers (Nicetto et al., 2019). The triple knockout results in a significantly different transcriptional profile compared to both the SETDB1 conditional knockout and wild type mouse liver cells (Nicetto et al., 2019). SETDB1 plays non-redundant roles in the silencing of the ERV family class I and class II long terminal repeat (LTR)-containing viruses (Matsui et al., 2010; Karimi et al., 2011; Collins et al., 2015; Fasching et al., 2015; Takikita et al., 2016; Kato et al., 2018; Adoue et al., 2019; Wang et al., 2020; Južnić et al., 2021). Widespread reactivation of ERVs in SETDB1 knockout cells also produces chimeric ERV-initiated transcripts that splice with genic exons and likely interfere with the expression of the native open reading frames (Karimi et al., 2011). G9a is essential for H3K9 methylation outside of pericentric heterochromatin and telomeres/subtelomeric domains along with some repetitive elements including rDNA repeats and class III ERVs (Tachibana et al., 2001; Tachibana et al., 2002; Tachibana et al., 2005; Collins et al., 2008; Maksakova et al., 2013; Jiang et al., 2020; Zhou et al., 2020). G9a and GLP display some overlap in function with SETDB1 when it comes to methylating and silencing intracisternal A-particles (IAPs) (Maksakova et al., 2013; Liu et al., 2014; di Giacomo et al., 2014). The role of SETDB2 in the silencing of repetitive elements is unknown. Further investigation of targets and functions of these methyltransferases in different cell types and contexts is required to better understand how H3K9me contributes to disease and developmental processes. In this section we will focus on the different types of repetitive DNA sequences that form constitutive heterochromatin.

### 2.1 Centromere and pericentromere

Centromeric chromatin is the site of kinetochore assembly during mitosis (Sullivan et al., 2001; Mellone and Allshire, 2003). Since histone H3 is replaced with the centromere-specific H3 variant CENP-A in centromeric heterochromatin, centromeres themselves lack H3K9 methylation (Yoda et al., 2000; Blower et al., 2002; Sullivan and Karpen, 2004). However, flanking pericentromeric chromatin is highly enriched in H3K9me2, H3K9me3, and heterochromatin protein 1 (HP1), which are all required for *de novo* CENP-A deposition at centromeres, proper microtubule attachment to kinetochores, sister chromosome cohesion, and chromosome segregation (Ekwall et al., 1995; Ekwall et al., 1996; Bernard et al., 2001; Nonaka et al., 2002; Folco et al., 2008; Yamagishi et al., 2008). Loss of either H3K9 methyltransferases or HP1 proteins cause chromosome segregation defects including micronuclei formation, chromosomal breaks and rearrangements, and aneuploidy (Ekwall et al., 1995; Ekwall et al., 1996; Peters et al., 2001; Peng and Karpen, 2009; Janssen et al., 2011; Crasta et al., 2012).

### 2.2 Telomere and subtelomere

Telomeres are specialized chromatin structures that cap and safeguard the ends of linear chromosomes (van Steensel et al., 1998; Karlseder et al., 2004; Yang et al., 2005; Bae and Baumann, 2007; Denchi and de Lange, 2007; Sfeir and de Lange, 2012). They are comprised of G-rich short tandem repeats that can reach as long as 50 kb long in mammals (Barral and Déjardin, 2020). These sequences are recognized by the telomere repeat-specific binding proteins of the Shelterin complex, which recruits telomerase and other factors that prevent an aberrant DNA damage response (van Steensel et al., 1998; Karlseder et al., 1999; Li and de Lange, 2003; Karlseder et al., 2004; Ye et al., 2004; Kelleher et al., 2005; Yang et al., 2005; Bae and Baumann, 2007; Denchi and de Lange, 2007; Wang et al., 2007; de Lange, 2018; Barral and Déjardin, 2020). Loss of Shelterin results in catastrophic chromosomal fusion where chromosomes become joined through their telomeres (van Steensel et al., 1998). Telomere lengthening is catalyzed by telomerase and can also occur through the telomerase-independent Alternative Lengthening of Telomeres (ALT) pathway, which is often associated with cancer (Dunham et al., 2000). This pathway enlists components of homologous recombination pathways to lengthen telomeres (Dunham et al., 2000). In *S. pombe*, both Clr4/Suv39 and the histone deacetylase Snf2/HDAC-containing Repressor Complex (SHREC) are recruited to telomeres by Shelterin, which subsequently establish heterochromatin in the adjacent subtelomeric repeats (Sugiyama et al., 2007; Zhang et al., 2008). The RNAi pathway also recruits Swi6/HP1 and SHREC to the subtelomeres and contributes to heterochromatin formation, however, this only occurs in *S. pombe* (Volpe et al., 2002; Zofall and Grewal, 2006; Déjardin and Kingston, 2009; Saksouk et al., 2014; Gauchier et al., 2019). In mammals, telomeric and subtelomeric chromatin is heterochromatic and the loss of silencing can cause aberrant recombination and DNA damage, but recent evidence suggests that this may only be true when the ALT pathway is activated (Lovejoy et al., 2012; Arora et al., 2014; Cubiles et al., 2018).

### 2.3 Transposable elements

Transposable elements (TE) make up at least 45% of the human genome with LINE-1 elements alone accounting for 17% of the genome (Cordaux and Batzer, 2009). Although most TEs lack transposition activity due to acquired inactivating mutations, some remain intact (Cordaux and Batzer, 2009). Silencing of TEs is required to prevent DNA damage caused by deleterious RNA:DNA structures and gene disruption or rearrangements caused by hopping mobile elements (Slotkin and Martienssen, 2007; Zeller et al., 2016). More than 1,200 distinct types of TEs have been identified (Kojima, 2018). For most of these, how they are regulated is unknown. However, several major classes including ERV class I elements, LINE elements, and major satellite repeats (MSRs) are all derepressed in the absence of H3K9me2 and H3K9me3 in mouse embryonic fibroblasts (Montavon et al., 2021).

## 3 Facultative heterochromatin and the regulation of protein-coding genes

**FIGURE 4:**
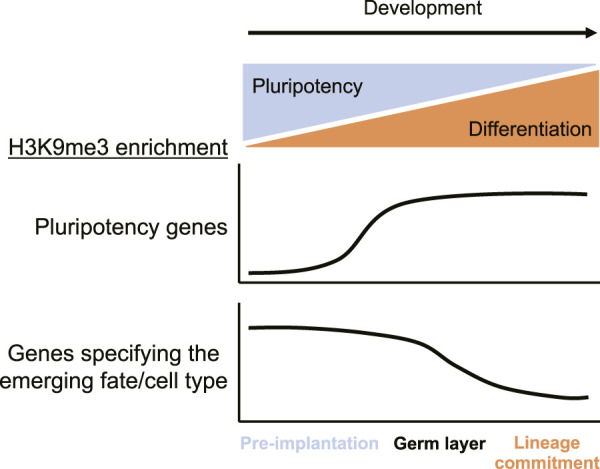
H3K9me3 enrichment at pluripotency versus cell type-specific genes throughout development. In early, pre-implantation stages, H3K9me3 is missing from pluripotency genes but highly enriched at cell type-specific genes, which promotes the pluripotency program. As development proceeds into the germ layer stage, H3K9me3 is deposited at pluripotency genes and becomes significantly lost at cell type-specific genes during lineage commitment to promote tissue specificity. H3K9me3 differentially regulates pluripotency and cell type-specific genes to orchestrate the timely progression of developmental processes.

Traditionally, H3K9me3 and H3K27me3 have been associated with constitutive and facultative heterochromatin, respectively. However, a more dynamic role for H3K9me3-mediated gene regulation during development has emerged, which challenges this classical view (Becker et al., 2017; Nicetto et al., 2019). Embryos with tissue-specific triple knockouts of SETDB1, SUV39H1, and SUV39H2 have a loss of cell type-specific gene expression programs in addition to derepression of lineage-inappropriate genes, leading to perturbation of cell identity (Nicetto et al., 2019). Although H3K9me3 levels are diluted over the first two to three cell divisions due to the lack of *de novo* H3K9me deposition, it becomes enriched at many protein-coding genes and constitutive heterochromatin sites during cell fate determination, which prevents premature activation of cell type-specific genes (Liu et al., 2004; Puschendorf et al., 2008; Nicetto et al., 2019). H3K9me undergoes dramatic reprogramming in the early stages of development with lineage-specific H3K9me3 patterns arising post-implantation (Wang et al., 2018). Upon differentiation, cell type-specific genes are derepressed through loss of H3K9me3 while lineage-inappropriate genes maintain H3K9me3 and transcriptional silencing (Figure 4) (Nicetto et al., 2019). Similar H3K9me dynamics have recently been reported in *C. elegans.* Genes expressed in embryos tend to gain H3K9me2/3 in differentiated cells, while cell type-specific genes that are expressed in differentiated cells tend to lose H3K9me2/3 (Methot et al., 2021). Together these studies demonstrate that H3K9me mediated gene silencing is specific to both cell type and developmental stage. In this section, we explore the role of H3K9me in facultative heterochromatin and its contribution to development.

In *S. pombe*, H3K9me2 plays a critical role in promoting vegetative growth and adaptation to environmental challenges (Zofall et al., 2012; Torres-Garcia et al., 2020). Vegetative growth is achieved through the formation of heterochromatin domains that silence meiotic genes including *mei4* and *ssm4* that are involved in cell cycle progression and microtubule organization during meiosis, respectively (Zofall et al., 2012). Nitrogen depletion triggers removal of H3K9me2 and derepression of these genes, which results in a shift from vegetative to sexual growth (Zofall et al., 2012). When returned to nitrogen-containing media, H3K9me2 is deposited at these meiotic genes and the cells resume vegetative growth (Zofall et al., 2012). H3K9me2 also mediates phenotypic plasticity in response to unfavourable growth conditions (Torres-Garcia et al., 2020). Caffeine is cytotoxic to *S. pombe* (Fabre, 1972; Loprieno et al., 1974; Gentner and Werner, 1975; Osman and McCready, 1998). However, exposure to threshold levels of caffeine causes resistance through the formation of epimutations or heritable changes in gene expression that do not affect the underlying DNA sequence (Torres-Garcia et al., 2020). Heterochromatin islands form over and reduce expression of distinct genes, some of which confer caffeine resistance when mutated (Torres-Garcia et al., 2020). The Mst2 histone acetyltransferase and Epe1, a putative H3K9 demethylase, cooperate to prevent the formation of heterochromatin islands (Wang et al., 2015). However, caffeine exposure reduces the levels of Epe1 and produces a shorter and likely functionally impaired isoform of Mst2 (Torres-Garcia et al., 2020). Thus, H3K9me2 heterochromatin is critical for the regulation of sex determination in *S. pombe* by modulating meiotic gene expression to promote a vegetative state and for phenotypic plasticity by conferring transient resistance to environmental challenges.

In mammals, H3K9me3 regulates genes involved in pluripotency to terminate stemness and facilitate differentiation (Feldman et al., 2006; Epsztejn-Litman et al., 2008). Pre-implantation, pluripotency factors lack H3K9me3 and are expressed, which facilitates stem-like properties (Figure 4) (Feldman et al., 2006; Epsztejn-Litman et al., 2008). However, following embryonic implantation, both pluripotency genes and lineage-specific genes are methylated at H3K9 and transcriptionally repressed (Figure 4) (Feldman et al., 2006; Epsztejn-Litman et al., 2008). The promoter region of murine *Oct-3/4*, for instance, is dynamically regulated by H3K9me during the pre- and post-implantation stages (Feldman et al., 2006). During pre-implantation, embryonic stem cells maintain high levels of the active H3K9 acetylation mark at the *Oct-3/4* gene, promoting pluripotency (Feldman et al., 2006). Retinoic acid-induced differentiation causes H3K9 deacetylation and G9a-mediated H3K9me3 deposition at the *Oct-3/4* locus, which facilitates transcriptional repression (Feldman et al., 2006). Similarly, H3K9me silences genes involved in stemness and memory during terminal differentiation of mouse CD8^+^ T cells to prevent reprogramming into pluripotent memory cells (Pace et al., 2018). In the nervous system, knocking out SUV39H1 and SUV39H2 in the adult hippocampus increases the proportion of progenitor cells relative to mature neurons and leads to high levels of progenitor proliferation *in vitro* (Guerra et al., 2021). Lysine-to-methionine mutations have the ability to globally reduce methylation levels at the corresponding lysine by interfering with SET domain methyltransferases (Lewis et al., 2013; Herz et al., 2014; Fang et al., 2016; Jayaram et al., 2016; Lu et al., 2016). Expression of the histone H3.3 variant with lysine 9 mutated to methionine (H3K9M) in mouse embryonic stem cells results in small embryoid bodies with reduced H3K9me3 levels, increased chromatin accessibility, continued expression of several pluripotency markers, and reduced expression of some markers of differentiation (Brumbaugh et al., 2019). Mice expressing H3K9M have increased multipotent progenitors and display a number of additional cell type-specific defects including aberrant lymphopoiesis and thrombocytosis (Brumbaugh et al., 2019). Interestingly, ceasing H3K9M expression reverses differentiation defects, at least in the case of *in vitro* B cell maturation (Brumbaugh et al., 2019). These studies highlight a role for H3K9me in the dynamic regulation of pluripotency genes at the onset of differentiation (Figure 4).

In addition to regulating pluripotency genes, H3K9me3 also prevents developmental relapse into a more primitive totipotent state by selectively silencing genes associated with the 2-cell stage (Wu et al., 2020). Reducing H3K9me in early development by knocking out SETDB1 leads to peri-implantation lethality, underscoring the importance of this methyltransferase in early embryogenesis (Dodge et al., 2004). The developmental transition from totipotency to pluripotency is marked by the upregulation of stem-cell factors including Oct4 and Nanog, and the downregulation of trophectoderm markers and genes involved in the 2-cell stage (Wu et al., 2020). Knocking out SETDB1 results in aberrant upregulation of Dux, a critical gene associated with 2-cell state totipotency, as well as markers of trophectoderm differentiation including Hand1 and Cdx2 (Bilodeau et al., 2009; Yuan et al., 2009; Lohmann et al., 2010; Wu et al., 2020). Thus, H3K9me deposition by SETDB1 at totipotency genes plays a critical role in regulating stem-like properties following the 2-cell stage. This suggests that H3K9me-mediated gene regulation is not only important for promoting the transition towards pluripotency, but also for preventing relapse into a totipotent state.

In later stages of development during differentiation, H3K9me2/3 regulates cell type-specific genes to promote cell identity and lineage commitment (Figure 4) (Guerra et al., 2021). After the epiblast stage, during germ layer specification in mice, protein-coding genes become significantly enriched in H3K9me3 (Figure 4) (Nicetto et al., 2019). Cell type-specific genes are dynamically derepressed through a loss of H3K9me3 after germ layer specification at the onset of organogenesis (Figure 4) (Nicetto et al., 2019). In endoderm cells, known markers of the hepatic lineage including the *Cyp* gene cluster are retained in H3K9me3-associated heterochromatin and become derepressed upon differentiation into hepatic progenitors and hepatocytes (Nicetto et al., 2019). However, genes associated with alternate cell fates, such as the pancreas-specific gene *Slc30a8*, remain H3K9me3-enriched and transcriptionally silent in differentiated hepatic cells (Nicetto et al., 2019). Furthermore, 2-month-old liver cells derived from endoderm-specific conditional triple knockout embryos for SETDB1, SUV39H1, and SUV39H2 have significantly reduced liver-specific gene expression and upregulation of lineage-nonspecific genes involved in various processes such as embryonic morphogenesis, heart development, and RNA processing/translation (Nicetto et al., 2019). Similarly, many genes expressed in *C. elegans* embryos gain H3K9me2/3 in differentiated cells, while cell type-specific genes that are expressed in differentiated cells lose H3K9me2/3 (Methot et al., 2021). Once established, these methylation patterns require active maintenance by at least the MET-2 SETDB1-like H3K9me1/2 methyltransferase (Methot et al., 2021). Although H3K9me2 blocks transcription factor binding and is necessary for silencing, its loss in MET-2 and SET-25 methyltransferase deficient animals is not sufficient for chromatin decompaction or gene activation (Methot et al., 2021). The presence of specific transcription factors is required for the expression of genes that lack H3K9me2/3, which helps to explain the observed cell type-specific effects of H3K9me2/3 on gene expression in *C. elegans* (Methot et al., 2021). H3K9me3 is also important for maintaining T_H_2 lymphocytes since SUV39H1 participates in T_H_1-specific gene repression and SETDB1 is required for maintaining stable lineage commitment (Allan et al., 2012; Adoue et al., 2019). SETDB1-dependent H3K9me3 also represses adipogenic master regulatory genes until differentiation is required and regulates cell fate decisions in murine neurogenesis, myogenesis, and oligodendrocyte differentiation (Tan et al., 2012; Liu et al., 2015; Matsumura et al., 2015; Beyer et al., 2016; Jiang et al., 2017). Loss of G9a during myogenesis and haematopoiesis results in derepression of lineage inappropriate genes and in the case of myogenesis, cell cycle regulators are also derepressed (Chen et al., 2012; Rao et al., 2016). G9a is also required for neuronal differentiation and the maintenance of the differentiated neurons (Fiszbein et al., 2016). Therefore, H3K9me3 deposition during the germ layer stage is essential for ensuring cell identity in later stages of differentiation and lineage commitment (Nicetto et al., 2019). During differentiation, H3K9me3 is dynamically lost at cell type-specific genes to promote their derepression while lineage-inappropriate genes retain H3K9me3 heterochromatin, which ensures proper cell fate determination (Figure 4) (Nicetto et al., 2019).

Once cells reach a terminally differentiated state, H3K9me3 forms a barrier to cellular reprogramming by maintaining gene expression programs that are critical for cell identity (Becker et al., 2016). Large domains of H3K9me3 coat important pluripotency genes in differentiated cells and hinder the binding of the Yamanaka transcription factors—Oct4, Sox2, Klf4, and c-Myc (Soufi et al., 2012). Suppressing H3K9 methyltransferases improves the efficiency of iPS cell generation by increasing the binding of Oct4 and Sox2 to these sites (Shi et al., 2008; Onder et al., 2012; Soufi et al., 2012; Sridharan et al., 2013). Similarly, SCNT is also enhanced when H3K9 methyltransferases are depleted or an H3K9 demethylase is expressed in conjunction with deacetylase inhibitor treatment (Matoba et al., 2014; Liu et al., 2018). Therefore, H3K9me not only plays important roles in establishing cell type-specific gene expression programs, but also in maintaining the stability of lineage commitment and cell identity.

Selective loss of H3K9me3 at appropriate genes regulates the timely progression of development from the 2-cell stage through lineage commitment. In early murine development, pluripotency genes lack H3K9me3 and are expressed (Figure 4). Pluripotency and cell type-specific genes are repressed by H3K9me3 during the germ layer stage, however the mechanism of cell type-specific gene silencing prior to germ layer specification is still unclear (Nicetto et al., 2019). In later stages of development during differentiation and lineage commitment, H3K9me3 ensures tissue-specific gene expression programs and cell identity through selective loss at cell type-specific genes and retention at lineage-inappropriate genes (Nicetto and Zaret, 2019). Taken together, these studies highlight the importance of H3K9me in development and cell fate determination.

## 4 Additional methylation targets

Although the best-known target of H3K9 methyltransferases is histone H3K9, they also regulate a growing list of additional histone and non-histone proteins through methylation and in some cases automethylation (for a comprehensive list see Table 1). In this section we provide an overview of automethylation, some key histone and non-histone targets of H3K9 methyltransferases, and outline the known biological consequences of these methylation events.

**TABLE 1:** Summary of additional histone and non-histone targets of H3K9 methyltransferases.

	Protein	Site	Function	Reference
Clr4 (*S. pombe*)	Clr4	K455, K472	Alleviates autoinhibition thereby enhancing activity of Clr4	Manzur and Zhou (2005)
	Mlo3	K167	Facilitates centromeric siRNA production	Paik et al. (2007)
G9a	Acinus	K654	-	Shi and Tsukada (2013)
	ATF7IP	K16	Induces transgene silencing by recruiting MPP8	Rea et al. (2000)
	CDYL1	K135	Decreases the interaction between CDYL1 and H3K9me3	Shi and Tsukada (2013)
	C/EBPβ	K39	Abrogates transactivation potential	Czermin et al. (2001)
	CSB	K170, K297, K448, K1054	-	Shi and Tsukada, (2013)
	DNMT1	K70	-	Shi and Tsukada (2013)
	DNMT3	K47	Creates a binding site for MPP8	Nakayama et al. (2001)
	ERα	K235	Induces transcription by recruiting the PHF20/MOF HAT complex	Kornberg (1974)
	Foxo1	K273	Induces Foxo1 degradation	Luger et al. (1997)
	G9a	K185, K239	Creates a binding site for HP1 proteins	Heitz (1928), Minc et al. (1999), Allis and Jenuwein (2016)
	GLP	K205	Creates a binding site for HP1 proteins and MPP8	Nakayama et al. (2001), Heitz (1928)
	H1.0	-	-	Horsley et al. (1996)
	H1.2	K187	-	Horsley et al. (1996)
	H1.3	-	-	Horsley et al. (1996)
	H1.4	K26	Creates a binding site for HP1 and L3MBTL1 proteins	Horsley et al. (1996), Ikegami et al. (2010)
	H1.5	-	-	Horsley et al. (1996)
	H3	K27	-	Pickersgill et al. (2006), Guelen et al. (2008)
	H3	K56	Involved in DNA replication	Peric-Hupkes et al. (2010)
	HDAC1	K432	-	Shi and Tsukada, (2013)
	HIF-1α	K674	Inhibits HIF-1α transcriptional activity	Funabiki et al. (1993)
	Lig1	K126	Recruits UHRF1 to replication sites	Poleshko et al. (2013)
	MDC1	K45	Promotes interaction with ATM at sites of DNA damage	Wong et al. (2014)
	MEF2D	K267	Inhibits MEF2D transcriptional activity	Solovei et al. (2013)
	MTA1	K532	Inhibits transcription by promoting assembly and recruitment of the co-repressor NuRD complex	Towbin et al. (2012)
	MyoD	K104	Inhibits MyoD transcriptional activity	Olins et al. (2010)
	p53	K373	May inhibit p53-dependent apoptosis	Brachner and Foisner, (2011)
	Plk1	K209	Inhibits Plk1 activity by antagonizing T210 phosphorylation	Larson et al. (2017)
	Pontin	K265, K267, K268, K274, K281, K285	Induces HIF-1α transcriptional activity by enhancing p300 recruitment	Strom et al. (2017)
	Reptin	K67	Inhibits HIF-1α transcriptional activity	Allshire and Madhani (2018)
	Sirt1	K622		Janssen et al. (2018)
	WIZ	K305	-	Shi and Tsukada (2013)
GLP	ATF7IP	K16	Induces transgene silencing by recruiting MPP8	Rea et al. (2000)
	DNMT3	K47	Creates a binding site for MPP8	Nakayama et al. (2001)
	ERα	K235	Induces transcription by recruiting the PHF20/MOF HAT complex	Kornberg (1974)
	GLP	K205	Creates a binding site for HP1 proteins and MPP8	Nakayama et al. (2001), Heitz (1928)
	H1.0	-	-	Horsley et al. (1996)
	H1.2	K187	-	Horsley et al. (1996)
	H1.3	-	-	Horsley et al. (1996)
	H1.4	K26	Creates a binding site for HP1 proteins	Horsley et al. (1996)
	H1.5	-	-	Horsley et al. (1996)
	H3	K27	-	Guelen et al. (2008)
	HIF-1α	K674	Inhibits HIF-1α transcriptional activity	Funabiki et al. (1993)
	Lig1	K126	Recruits UHRF1 to replication sites	Poleshko et al. (2013)
	MDC1	K45	Promotes interaction with ATM at sites of DNA damage	Wong et al. (2014)
	p53	K373	May inhibit p53-dependent apoptosis	Brachner and Foisner (2011)
	Pontin	K265, K267, K268, K274, K281, K285	Induces HIF-1α transcriptional activity by enhancing p300 recruitment	Strom et al. (2017)
SETDB1	AKT	K64, K140, K142	Activates AKT, promoting localization to the cell membrane and tumorigenesis	Soufi et al. (2012), Onder et al. (2012)
	p53	K370	May increase mutant p53 stability	Shi et al. (2008)
	Tat	K50, K51	Inhibits ternary complex formation with Cdk9/cyclin T and the TAR RNA	Sridharan et al. (2013)
SUV39H1	DOT1L	K410	-	Liu et al. (2018)
	HupB	K138	-	Matoba et al. (2014)
	RAG2	K507	Alters RAG2 subnuclear localization	Liu et al. (2018)
	SET8	K210	Stimulates SET8 activity	Liu et al. (2018)
SUV39H2	LSD1	K322	Reduces LSD1 degradation	Montavon et al. (2021)
	SUV39H2	K392	Impairs binding affinity to target proteins including H3 and LSD1	Fritsch et al. (2010)

### 4.1 Histone targets

G9a has been suggested to methylate other sites on histone H3 including H3K27 and H3K56. Both G9a and GLP methylate H3K27 *in vitro* and in embryonic stem cells loss of G9a reduces H3K27me1 levels by 30% (Tachibana et al., 2001; Wu et al., 2011). H3K56me1 interacts directly with PCNA *in vitro* and the two colocalize in the G1 phase of the cell cycle (Yu et al., 2012). Knockout or depletion of G9a or mutating H3K56 impairs DNA replication (Yu et al., 2012). In addition to canonical histone proteins that form the proteinaceous core of the nucleosome, linker histones are another major component of chromatin (Woodcock et al., 2006). Humans have 11 different H1 variants, five of which are methylated by G9a and GLP (Trojer et al., 2009; Weiss et al., 2010; Brockers and Schneider, 2019). G9a and GLP methylate H1.4 on its N-terminus at lysine 26, which can be reversed by the KDM4 demethylase (Trojer et al., 2009; Weiss et al., 2010). H1.4K26me forms a binding site for HP1 proteins, which suggests that this modification may have a role in transcriptional gene silencing and heterochromatin formation (Trojer et al., 2009; Weiss et al., 2010). G9a and GLP methylate H1.0, H1.2, H1.3, and H1.5 on their C-terminus (Weiss et al., 2010). However, unlike H1.4K26me, H1.2K187me does not bind HP1 proteins, which reveals H1 variant-specific functions of G9a and GLP methylation that require further investigation (Trojer et al., 2009; Weiss et al., 2010).

### 4.2 Non-histone targets

#### 4.2.1 SUV39H2 and Clr4 automethylation

**FIGURE 5:**
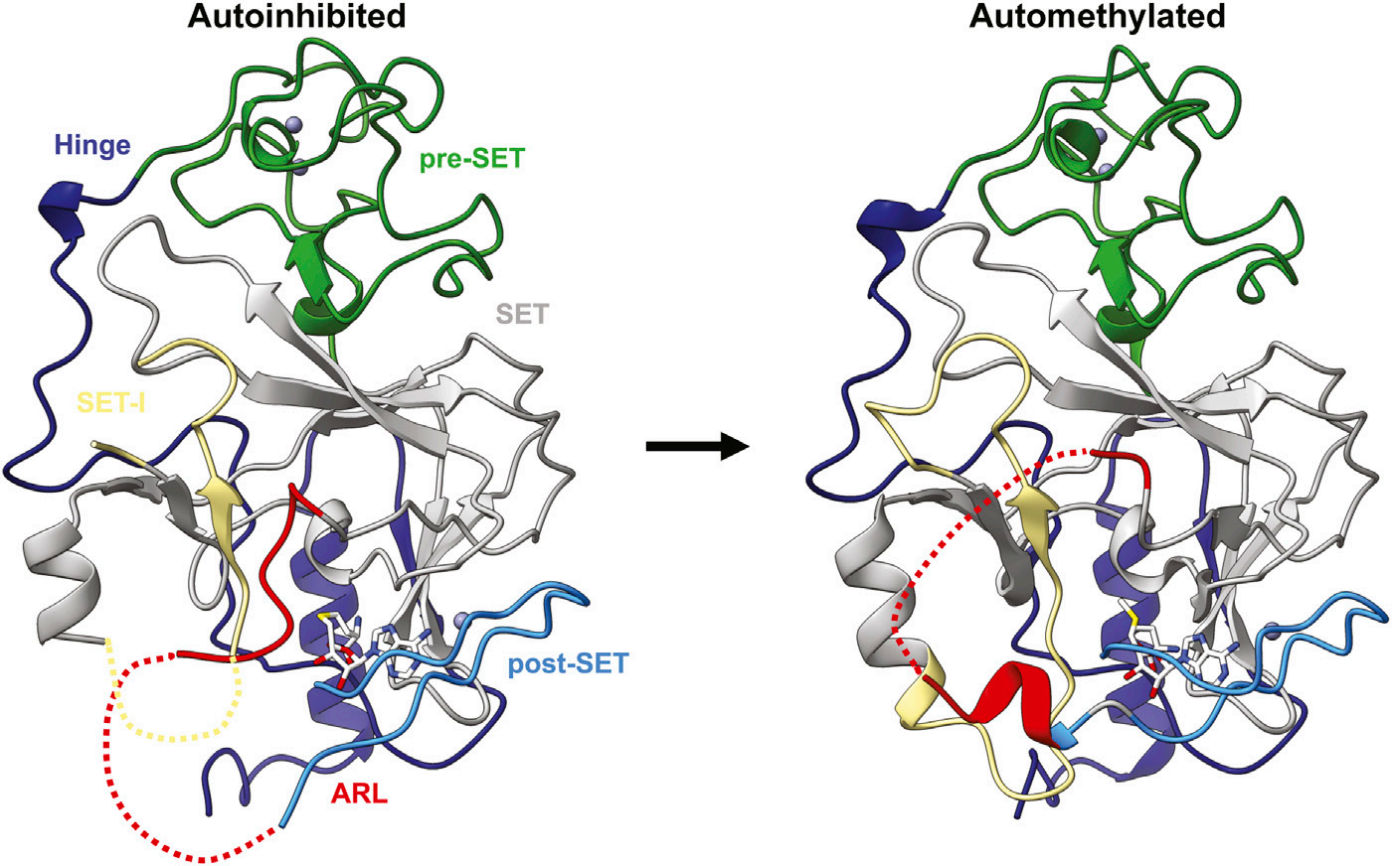
Clr4 automethylation-induced conformational switch. Structure of the autoinhibited (left; PDB ID 6BOX) and automethylated (right; PDB ID 6BP4) Clr4 catalytic domains with domains coloured the same as Figure 1 with the autoregulatory loop coloured red.

Formation of repressive chromatin domains is tightly regulated to prevent deleterious epigenetic gene silencing. This regulation occurs at many levels and includes pathways that recruit H3K9 methyltransferases, extrinsic antisilencing factors such as H3K9 demethylases, and mechanisms of histone turnover. However, it was recently shown that the activity of Clr4 itself is regulated through a novel intrinsic autoregulatory mechanism (Iglesias et al., 2018). An internal loop, dubbed the autoregulatory loop (ARL) in Clr4, inhibits enzyme activity by blocking the substrate-binding pocket (Figure 5) (Iglesias et al., 2018). Intramolecular automethylation of two lysines within the loop, K455 and K472, promotes a conformational switch in the enzyme that opens the substrate-binding pocket and enhances Clr4 activity (Figure 5) (Iglesias et al., 2018). Mutating Clr4 automethylation sites *in vivo* disrupts this autoregulation, resulting in aberrant H3K9me2 and H3K9me3, loss of heterochromatin domains, and slow growth in *S. pombe* (Iglesias et al., 2018). This demonstrates the critical role Clr4 autoregulation plays in both regulating H3K9me2 and H3K9me3 deposition and maintaining epigenetic stability. The second more C-terminal automethylation site, K472, is broadly conserved within the SUV39H family of methyltransferases (Iglesias et al., 2018). However, the first automethylation site, K455, is only found in the mammalian SUV39H2 enzyme and corresponds to K392 (Iglesias et al., 2018). This site is automethylated both *in vitro* and *in vivo* and impairs binding to substrates histone H3 and LSD1 *in vitro* (Piao et al., 2016). However, whether SUV39H2 automethylation plays the same regulatory role as Clr4 in mammalian cells has not been explored.

#### 4.2.2 SUV39H1, SUV39H2, and Clr4 non-histone targets

Clr4 interacts with the RNA-induced transcriptional silencing (RITS) complex, a central component of the RNAi pathway in *S. pombe*, and the RNA processing and export factor Mlo3 (Zhang et al., 2008; Bayne et al., 2010; Zhang et al., 2011). These interactions bridge heterochromatin formation and RNA processing by channeling antisense and centromeric RNAs into the RNAi pathway. In addition to binding Mlo3, Clr4 also methylates Mlo3 on K167 (Zhang et al., 2011). Abrogating this methylation *in vivo*, by mutating K167 and adjacent K165 to alanine, impairs Mlo3 function causing a decrease in centromeric siRNA levels and weak upregulation of antisense RNAs (Zhang et al., 2011). Mlo3 methylation may influence the recognition of aberrant RNA by other factors, such as the RITS complex, however, this hypothesis has not been tested (Zhang et al., 2011). Ultimately, how Clr4-mediated methylation of Mlo3 contributes to Mlo3 function remains unclear.

Mammalian SUV39H1 and SUV39H2 also target a variety of non-histone proteins for methylation, specifically chromatin regulatory factors involved in reading, writing, or erasing alternate histone marks. SUV39H1 regulates the subnuclear localization of RAG2, an H3K4me3 reader that functions in variable diversity joining, or VDJ, recombination, via methylation of RAG2 K507 (Kudithipudi et al., 2017). Wild type and K507R mutant RAG2 both display speckled nuclear distribution when expressed in NIH3T3 cells (Kudithipudi et al., 2017). When coexpressed with SUV39H1, wild type RAG2 loses this focal clustering and becomes uniformly distributed throughout the nucleus, while the localization of K507R mutant RAG2 remains unchanged (Kudithipudi et al., 2017). Coexpression of wild type RAG2 with a catalytically inactive version of SUV39H1 also did not alter RAG2 distribution (Kudithipudi et al., 2017). This suggests that SUV39H1 methyltransferase activity may regulate RAG2-mediated VDJ recombination or its other functions.

In addition to H3K9me, other histone modifications are enriched in heterochromatin in mammals including H4K20me2, H4K20me3, H3K23me3, H3K56me3, and H3K64me3 (Schotta et al., 2004; Jack et al., 2013; Lange et al., 2013; Papazyan et al., 2014; Schwartz-Orbach et al., 2020). H4K20me2 and H4K20me3 are both produced by the methyltransferase SUV420H (Schotta et al., 2004). However, SUV420H requires the methyltransferase SET8 to deposit H4K20me1 first in order for the SUV420H enzymes to generate the two higher order methylation states, H4K20me2 and H4K20me3 (Schotta et al., 2004; Karachentsev et al., 2005; Schotta et al., 2008). SUV39H1 also contributes to H4K20 methylation by regulating SET8 activity (Kudithipudi et al., 2017). SUV39H1 methylates K210 of SET8 *in vitro*, which enhances SET8 methyltransferase activity (Kudithipudi et al., 2017). Thus, methylation of SET8 may represent another important contribution of SUV39H1 to heterochromatin formation in addition to producing H3K9me2 and H3K9me3.

LSD1 is a histone demethylase that promotes gene silencing by erasing the transcriptionally active H3K4me1 and H3K4me2 marks (Piao et al., 2015). SUV39H2 regulates this function by methylating LSD1 K322 (Piao et al., 2015). However, the mechanism of regulation differs from that of SET8 regulation by SUV39H1 methylation. Instead of enhancing enzyme activity, methylation of LSD1 by SUV39H2 increases its stability by inhibiting polyubiquitination and subsequent degradation by the proteasome (Piao et al., 2015). This is another example of non-histone target methylation by a SUV39-family enzyme that complements its role in heterochromatin function. In this case, SUV39H2 both deposits the silencing marks H3K9me2 and H3K9me3 and facilitate the removal of active marks, H3K4me1 and H3K4me2, by stabilizing the LSD1 demethylase (Piao et al., 2015).

#### 4.2.3 G9a and GLP automethylation

Like SUV39H2 and Clr4, G9a and GLP also possess automethylation activity and automethylate motifs that resemble histone H3 at K185 and K239, for G9a, and K205, for GLP (Chin et al., 2007; Sampath et al., 2007; Poulard et al., 2017). Automethylation of these histone mimics creates a binding site for HP1 family proteins, which can be blocked by phosphorylation of the subsequent threonine, T186 in G9a and T206 in GLP, by the Aurora B kinase (Chin et al., 2007; Sampath et al., 2007; Poulard et al., 2017). This regulation is reminiscent of HP1 eviction from their H3K9me marked binding sites during mitosis by Aurora B mediated phosphorylation of H3S10 (Fischle et al., 2005). In addition to their roles in heterochromatin formation and gene silencing, G9a and GLP also form complexes that are responsible for gene activation (Poulard et al., 2018). The ternary complex of G9a or GLP with HP1γ and Glucocorticoid Receptor (GR) upregulates a subset of GR target genes (Poulard et al., 2018). Inhibition of JmjC family lysine demethylases, using the small molecule inhibitor JIB-04, increases HP1γ and GR complex formation and expression of GR target genes that are upregulated by G9a, GLP, and HP1γ likely due to enhanced G9a methylation stabilizing HP1γ binding (Poulard et al., 2018). A screen of lysine demethylases demonstrated that G9a automethylation can be removed by KDM4 *in vitro* (Poulard et al., 2018). Therefore G9a-HP1γ complex formation and its role in transcriptional regulation of GR target genes can be regulated dynamically by reversible methylation and phosphorylation.

#### 4.2.4 G9a and GLP non-histone targets

Just like G9a automethylation produces a new binding site to recruit additional chromatin factors, so does G9a-mediated methylation of other non-histone targets. G9a methylates oestrogen receptor α (ERα), a nuclear hormone receptor that mediates the cells response to oestrogen, at K235 (Zhang et al., 2016). This methylation is recognized by the tandem tudor domain of PHF20, which recruits the MOF histone acetyltransferase complex to ERα target genes where it promotes transcription by depositing H4K16 acetylation (Zhang et al., 2016). DNA ligase 1 (LIG1) is methylated by G9a and GLP on a sequence that resembles histone H3K9 (Ferry et al., 2017). This creates a binding site for UHRF1, which recruits UHRF1 to replication sites (Ferry et al., 2017). Disruption of this interaction results in a significant reduction of DNA methylation in mouse embryonic stem cells (Ferry et al., 2017). ATF7IP is also methylated by G9a and GLP on a histone H3K9 mimic, which forms a binding site for the chromodomain of M-phase phosphoprotein 8 (MMP8), a component of the human silencing hub (HUSH) complex (Tsusaka et al., 2018). Expression of an unmethylatable mutant version of ATF7IP impairs SETDB11/MPP8-dependent silencing in a provirus reporter silencing assay (Tsusaka et al., 2018).

G9a also regulates several TFs through methylation. G9a methylates a conserved lysine (K39) in the transactivation domain of C/EBPβ, a basic leucine zipper TF that regulates tissue-specific gene expression, cell proliferation, and differentiation (Pless et al., 2008). C/EBPβ methylation suppresses its transcription activity (Pless et al., 2008). G9a also regulates a number of TFs involved in skeletal muscle differentiation. Myogenesis-promoting TF MyoD is methylated on K104, which inhibits transcription activity and suppresses myogenic differentiation (Ling et al., 2012). G9a mono- and dimethylates MEF2 on K267, which suppresses MEF2 transcription activity and downregulates genes important for myogenesis (Choi et al., 2014). G9a-mediated methylation of MEF2 has also been suggested to inhibit p38α-mediated phosphorylation of MEF2 at residues adjacent to K267 (Choi et al., 2014). Since phosphorylation of MEF2 by p38α promotes its transcription activity, G9a-mediated methylation may represent an inhibitory mechanism that impedes MEF2 phosphorylation to prevent aberrant gene activation.

Additionally, G9a methylates a variety of non-histone proteins that are involved in the hypoxia stress response pathway. Pontin is a chromatin remodeling factor involved in regulating the hypoxia response, which is methylated by G9a and GLP at several sites (Lee et al., 2011). Under normal conditions, Pontin displays low basal levels of methylation (Lee et al., 2011). Hypoxic conditions strongly induce methylation of Pontin *in vivo* and promote the expression of Pontin-dependent hypoxia target genes (Lee et al., 2011). A mutant version of Pontin with all lysine methylation sites replaced with alanine (K265A, K267A, K268A, K274A, K281A, and K285A) no longer displays hypoxia-induced methylation and fails to activate a subset of Pontin target genes (Lee et al., 2011). This Pontin mutant also impairs proliferation and migration within the MCF7 breast cancer cell line (Lee et al., 2011). G9a regulates another chromatin remodeling factor involved in the hypoxia stress response, called Reptin (Lee et al., 2010). G9a methylates Reptin at K67 under hypoxic conditions, which negatively regulates a subset of hypoxia response genes by suppressing HIF-1α transcriptional activity (Lee et al., 2010). HIF-1α, the master transcription regulator of the hypoxia response pathway, is also methylated by G9a at K674, which reduces downstream target gene activation (Bao et al., 2018). Combined, this reveals an important role for G9a and GLP non-histone target methylation in both positive and negative regulation of the hypoxia stress response pathway.

#### 4.2.5 SETDB1 non-histone targets

SETDB1-mediated non-histone methylation regulates several important pathways that contribute to disease. AKT kinase regulates metabolism, cell proliferation, and survival and its hyperactivation plays an important role in tumorigenesis (Manning and Toker, 2017). K63-linked ubiquitination of AKT is essential for both activation and relocation of the enzyme to the cell membrane (Yang et al., 2009; Chan et al., 2012). SETDB1 di- and trimethylates AKT on a lysine that is adjacent to this ubiquitination site, K64 (Wang et al., 2019). This forms a binding site for JMJD2A, which in turn recruits E3 ligase resulting in ubiquitinated AKT (Wang et al., 2019). K64 methylation stimulates AKT activity, localization to the cell membrane, and its prosurvival function, which promotes tumorigenesis and correlates with poor prognosis in non-small cell lung cancer patients (Wang et al., 2019). SETDB1 also trimethylates K140 and K142 of AKT (Guo et al., 2019). These additional methylation sites bind the tudor domain of SETDB1 reinforcing its interaction with AKT, enhancing K64 methylation, and ultimately AKT activity (Guo et al., 2019).

The tumour suppressor, p53, regulates the cell cycle, apoptosis, and genome stability (Oren, 2003). It is frequently mutated in cancer where a number of gain-of-function (GOF) mutations have been identified (Brosh and Rotter, 2009). SETDB1 methylates wild type p53 and at least one of its GOF mutants, R249S, on K370 (Fei et al., 2015). P53R249S displays increased stability relative to wild type p53, which is partially dependent on SETDB1 (Fei et al., 2015). However, whether this increased stability is caused by methylated K370 or SETDB1 is unclear. SETDB1 binds more tightly to the GOF mutant relative to wild type p53 (Fei et al., 2015). Therefore, the increased stability of GOF p53 could be attributed to SETDB1 binding instead of its methyltransferase activity or potentially both contribute.

SETDB1 may also regulate HIV viral pathogenesis. SETDB1 binds and methylates the HIV-1 Tat protein on K50 and K51 (van Duyne et al., 2008). The Tat protein forms a ternary complex with Cdk9/cyclin T and the TAR RNA molecule, which is disrupted by K50 and K51 methylation (van Duyne et al., 2008). Knockdown of SETDB1 increases activation of HIV-1 LTRs in two reporter systems and enhanced reverse transcriptase activity *in vivo* (van Duyne et al., 2008). However, the *in vivo* role K50 and K51 methylation plays in HIV pathogenesis remains to be tested.

## 5 Conclusion

The studies discussed here highlight the growing repertoire of genomic, histone, and non-histone targets of H3K9 methyltransferases. This has expanded the role of these methyltransferases beyond the maintenance of genome stability and the formation of constitutive heterochromatin. However, further investigation is needed to understand how these novel methylation events are regulated, when they are used by the cell, and how they integrate with the other functions of these enzymes. How H3K9 methyltransferases are recruited to sites of constitutive heterochromatin has been studied extensively. However, how H3K9me is selectively deposited and removed from protein-coding genes at different stages of development is not fully understood. Moreover, the molecular mechanisms that trigger and turn over non-histone methylation and automethylation also remain largely unknown. What we have summarized here likely only scratches the surface. A complete methylproteome for these methyltransferases will be required to appreciate the full scope of cellular, developmental, and pathological processes regulated by H3K9 methyltransferases.
